# VISCERAL SENSITIVITY INDEX (VSI-IT): Italian Adaptation and Validation

**DOI:** 10.3390/ejihpe14070130

**Published:** 2024-07-05

**Authors:** Amelia Rizzo, Aurora Mautone, Aldo Sitibondo, Gabriella Nucera, Livio Tarchi, Hicham Khabbache, Driss Ait Ali, Khalid Ouazizi, Łukasz Szarpak, Michal Pruc, Murat Yıldırım, Francesco Chirico

**Affiliations:** 1 Department of Cognitive Sciences, Psychological, Educational, and Cultural Studies, University of Messina, 98122 Messina, Italy; 2 Department of Clinical and Experimental Medicine, University of Messina, 98122 Messina, Italy; aurora.mautone.25@gmail.com; 3 Infectious Diseases Unit, University Hospital “G. Martino”, 98124 Messina, Italy; 4 Department of Emergency, Fatebenefratelli Hospital, ASST Fatebenefratelli and Sacco, 20121 Milan, Italy; gabriellanucera@gmail.com; 5 Department of Health Sciences, University of Florence, 50127 Florence, Italy; livio.tarchi@unifi.it; 6 Department of Psychology, Faculty of Arts and Human Sciences Fès-Saïss, Sidi Mohamed Ben Abdellah University, Fez 30050, Morocco; hichamcogn@gmail.com (H.K.); driss.aitali@usmba.ac.ma (D.A.A.); khalid.ouazizi@usmba.ac.ma (K.O.); 7 Department of Clinical Research and Development, LUXMED Group, 02-676 Warsaw, Poland; lukasz.szarpak@gmail.com (Ł.S.);; 8 Department of Psychology, Faculty of Science and Letters, Agri Ibrahim Cecen University, Ağrı 04100, Türkiye; muratyildirim@agri.edu.tr; 9 Department of Social and Educational Sciences, Lebanese American University, Beirut 1102-2801, Lebanon; 10 Post-Graduate School of Occupational Medicine, Faculty of Medicine and Surgery, Catholic University of the Sacred Heart, 00168 Rome, Italy; francesco.chirico@unicatt.it

**Keywords:** VSI, IBD, IBS, Crohn’s disease, ulcerative colitis, visceral sensitivity, visceral anxiety

## Abstract

The Visceral Sensitivity Index (VSI) represents a significant advancement in the assessment of gastrointestinal-specific anxiety among patients with irritable bowel syndrome (IBS) and chronic inflammatory bowel diseases (IBD)—such as ulcerative colitis and Crohn’s disease. However, an Italian version of the instrument is not yet available for the Italian-speaking population. This study utilized a national sample of 500 individuals divided into four groups: (a) patients with Crohn’s disease, (b) patients with ulcerative colitis, (c) patients with IBS, and (d) healthy controls (individuals without any diagnoses) to test the validity and reliability of the Italian VSI. Using back-translation methodology to ensure translation fidelity, this research applied a questionnaire and the VSI through an online format to 500 participants. Confirmatory Factor Analysis (CFA) revealed that the Italian VSI had excellent psychometric properties, demonstrating high internal consistency (Cronbach’s α = 0.949) and construct validity. The scale proved sensitive in detecting significant differences in visceral sensitivity among groups, highlighting its utility as a clinical and research assessment tool. Specifically, the Italian VSI exhibited a unidimensional factorial structure and maintained a strong correlation with interoceptive awareness, type of disease, and gastrointestinal symptom severity, confirming its role in enhancing the understanding and management of IBD and IBS in Italy.

## 1. Introduction

The Visceral Sensitivity Index (VSI) is a psychometric assessment tool developed to specifically measure visceral sensitivity and anxiety related to gastrointestinal disorders, particularly within the context of irritable bowel syndrome (IBS). This index is designed to better understand how patients with IBS perceive and react to gastrointestinal symptoms, which often include abdominal pain, bloating, and alterations in bowel habits. The VSI specifically focuses on symptom-related anxiety known as gastrointestinal-specific anxiety (GSA) [1,2]. This type of anxiety is characterized by fear and apprehension regarding visceral sensations and symptoms, which can significantly impact the patients’ quality of life.

Through 15 items, the VSI assesses the frequency and intensity of these concerns and how they influence the patient’s daily behavior. The validity and reliability of the VSI have been confirmed through various studies, showing a strong correlation with other measures of anxiety and sensitivity, as well as the severity of gastrointestinal symptoms [1,2]. This makes the VSI a useful tool for clinicians and researchers in identifying and addressing the psychological aspects of IBS, contributing to a more holistic and personalized approach to managing this complex condition [3].

The VSI was originally developed in English but, over time, it has been translated and validated in various other languages to adapt to different cultural and linguistic contexts. These translations enable healthcare professionals to use the VSI in a variety of populations, enhancing the understanding and treatment of IBS globally.

The initial version of the VSI was developed by Labus and colleagues in 2004 [1]. The research focused on creating a valid and reliable psychometric tool to measure symptom-specific anxiety related to gastrointestinal symptoms—an aspect considered significant in the pathophysiology and health outcomes of patients with IBS. In the process of developing the VSI, researchers engaged both external and internal experts, as well as a group of patients, to evaluate a broad set of potential items gathered from the psychological and gastrointestinal literature.

After administering these potential scale elements to 96 patients diagnosed with IBS along with a set of validation questionnaires, a final item selection was made based on rigorous empirical criteria. The result was a unidimensional scale of 15 items: the Visceral Sensitivity Index, which demonstrated excellent reliability and good content, convergent, divergent, and predictive validity [1].

Subsequently, Lind et al. [4] translated and validated the Norwegian version of the VSI. The validity study of the Norwegian version investigated whether psychological factors such as general and symptom-specific anxiety and depression could predict the severity of symptoms in patients with unexplained subjective food hypersensitivity. To do so, seventy consecutive patients completed questionnaires on the Hospital Anxiety and Depression Scale, VSI, Irritable Bowel Syndrome Symptom Questionnaire, and Subjective Health Complaints Inventory. Multiple regression analyses were used to study the relationship between psychological factor scores and the severity of somatic symptoms. The results showed that most patients reported non-gastrointestinal symptoms in addition to the typical complaints of irritable bowel syndrome, but general and symptom-specific anxiety and depression did not explain a significant portion of the variance in somatic complaints. Symptom-specific anxiety for gastrointestinal symptoms was a significant predictor of gastrointestinal complaints, and age was the only significant predictor of non-gastrointestinal complaints. About 90% of the total variance in symptom severity remained unexplained by psychological factors. The Norwegian version of the VSI demonstrated satisfactory validity with a Cronbach’s alpha of 0.93, and there was a significant correlation between symptom-specific anxiety and general anxiety [4].

Subsequently, the Japanese version of the Visceral Sensitivity Index (VSI-J) was validated in a study conducted by Tatsuo Saigo and colleagues in 2014 [5]. This study translated the VSI into Japanese and assessed its reliability and validity. The primary objective was to introduce a Japanese version of the VSI to measure symptom-specific anxiety related to gastrointestinal symptoms (GSA) in patients with IBS, considering the importance of GSA as an endpoint for therapeutic interventions. The participant group consisted of 349 university students aged 18 and 19 years. The study used the VSI-J along with other instruments such as the Anxiety Sensitivity Index (ASI), the Hospital Anxiety and Depression Scale (HAD), and the IBS Severity Index (IBS-SI) to analyze the internal consistency, stability, and factorial structure of the VSI-J, along with its associations with anxiety, depression, and measures of intestinal pathology severity. The factorial structure of the VSI-J was found to be unidimensional and similar to that of the original VSI, with high internal consistency (Cronbach’s α = 0.93). Construct validity was demonstrated by significant correlations with ASI, HAD-ANX, and IBS-SI scores. Additionally, the VSI-J proved to be a significant predictor of severity scores on the IBS-SI and showed good discriminant and incremental validity [5].

More recently, the validation of the Ukrainian version of the Visceral Sensitivity Index (VSI-UA) has represented a significant development in the field of gastroenterology, particularly for Ukrainian-speaking patients with IBS. The study conducted by Neverovskyi, Shypulin, and Mikhnova [6] translated and validated the VSI to adapt it to the linguistic and cultural needs of the Ukrainian population, demonstrating the effectiveness of the tool in assessing specific anxiety related to the gastrointestinal tract. The results of the study show that the VSI-UA possesses good psychometric properties, with high internal reliability indicated by a Cronbach’s alpha value of 0.84, and test–retest consistency confirmed by an Intra-Class Correlation (ICC) coefficient of 0.92. Furthermore, the VSI-UA demonstrated excellent content validity, with a Content Validity Index (CVI) of 0.94 and a Content Validity Ratio (CVR) exceeding the critical value for each item. Construct validity was also confirmed through moderate and positive correlations with validated instruments such as the Patient Health Questionnaire-9 (PHQ-9), Beck Depression Inventory (BDI), and the Hospital Anxiety and Depression Scale (HADS) [6].

In clinical settings, a pronounced focus on physical sensations is commonly associated with disorders such as anxiety, hypervigilance, somatization, and hypochondriasis [4]. This type of heightened interoceptive awareness is generally viewed as maladaptive and potentially harmful. Interoception, which involves the nervous system’s capacity to sense, decode, and integrate signals from within the body, has gained prominence as a crucial area of study in the context of mind–body interactions and psychosomatics [7,8]. Being a key construct in this field, interoceptive accuracy—recently also termed interoceptive sensitivity—plays a vital role in research into how individuals perceive internal bodily states [9].

Currently, there is no Italian version of the Visceral Sensitivity Index, a crucial tool for assessing GSA in IBS and inflammatory bowel diseases (IBD) patients [10,11]. In Italy, it is estimated that about 250,000 people suffer from chronic inflammatory bowel diseases [12]. Assessing GSA in the Italian population is a timely concern. In fact, in the last ten years, the incidence of new cases in Italy has increased about 20 times. In 2018, there were 15,141 prevalent IBD patients, corresponding to 442.3 per 100,000 inhabitants/year. The prevalence increased by approximately 10% annually from 2010, with projections for Italy estimating over 15,000 new cases/year [12]. The need for an Italian adaptation also arises from the importance of culturally and linguistically specific instruments in medical research and practice. Such an adaptation would enable more accurate assessment and management of IBS and IBD in Italian-speaking populations, facilitating a better understanding of the disorder’s impact on patients’ lives and improving treatment outcomes in a culturally relevant manner.

Considering these premises, the objectives of this present study can be summarized as follows:

RQ1. Investigate the reliability and validity of the VSI-IT (Visceral Sensitivity Index-Italian).

RQ2. Explore the sensitivity and discriminant validity of the VSI-IT.

RQ3. Examine the concurrent validity of the VSI-IT.

## 2. Materials and Methods

### 2.1. Procedure

After obtaining approval from the Ethics Committee at the IRB of the Polish Society of Disaster Medicine (approval date: 3 January 2023. Approval no. 15.01.2023.IRB), subjects were recruited online through social network groups created by ambassadors and patients who are members of AMICI-ONLUS. Additionally, recruitment took place in-person at the University of Messina. Prior to administration, all participants provided informed consent. Data collection occurred from 13 February 2023 to 23 March 2023.

The back translation process for the Italian version of the Visceral Sensitivity Index involved the initial translation of the text from English to Italian (See Appendix A) by an experienced translator, followed by a review from a professional in the field of gastroenterology (A.S.), to ensure that all terms and concepts were accurately interpreted. Subsequently, a second translator, a native English speaker, converted the Italian text back into English without seeing the original document. The two English versions were then compared to identify discrepancies, which were discussed and resolved with the help of bilingual experts. After necessary adjustments to refine the accuracy of the translation, the Italian version was pretested on a small sample to ensure its clarity and relevance. Finally, it was validated through comparison with the original version to confirm its conceptual and linguistic fidelity.

Through the informed consent form (containing information regarding data confidentiality and the option to discontinue participation at any time), each participant provided their consent before proceeding with the survey.

### 2.2. Participants

The study involved 500 Italian-speaking participants (Table 1), including 108 males, 391 females, and 1 non-binary individual, with an age range from 18 to 85 years (M = 35.04; SD = 13.29). The power calculation for sample size was performed for an experimental design aimed at detecting differences between means (continuous data). This calculation was predicated on the hypothesis of superiority (Alternative H1), with a Type I error rate (α) set at 5% (0.05). Utilizing a sample size of 500, the analysis yielded a power value of 0.9977, or 99.77%, indicating a very high ability to detect an absolute difference between the groups under study.

Patients were asked to self-disclose a diagnosis of (i) Crohn’s disease, (ii) ulcerative colitis, (iii) irritable bowel syndrome (IBS), (iv) undergoing further testing before reaching a diagnosis, or (v) none of the above. Afterwards, four groups were formed, including (a) 111 patients with Crohn’s disease (CD), (b) 180 with ulcerative colitis (UC), (c) 34 with IBS, and (d) a control group with healthy subjects (164 participants). From the original sample of 500 participants, 11 self-disclosed gastrointestinal symptoms amenable to IBS or IBD but were still awaiting a formal diagnosis.

The inclusion criteria were age above 18 years, being Italian-speaking, and having access to an internet-connected device.

The majority of participants resided in the south of Italy (39.9%), followed by residents in the north (28.1%), the islands (18.0%), and a smaller number in the central region (13.6%). There was a predominant response from a female sample (78.0%) and a partial response from a male audience (21.4%), with a minority identifying as non-binary (0.2%). Most participants reported being single (40.3%), followed by married (36.5%), cohabiting (17.8%), divorced (2.8%), separated (1.8%), and widowed (0.4%). The majority of participants had a higher education level (51.1%), followed by a bachelor’s degree (22.0%) and postgraduate studies (15.0%). A minority reported completing only middle school (7.6%), having specialized diploma (1.6%), or having a Ph.D. (1.6%), with a few participants having a three-year qualification (0.2%), a first-level university master’s degree (0.2%), or professional training courses in healthcare assistance (0.2%). Most participants identified as students (34.3%), followed by employees (28.1%), freelancers (10.0%), homemakers (10.0%), workers (7.6%), unemployed (3.6%), self-employed (2.2%), retirees (1.8%), interns/trainees (0.8%), doctoral candidates/university researchers (0.8%), and volunteers (0.4%).

### 2.3. Instruments

In this investigation, we employed the Visceral Sensitivity Index (VSI) and the Multidimensional Assessment of Interoceptive Awareness (MAIA) to examine interoception as a critical construct. The MAIA was selected for its ability to comprehensively measure multiple facets of interoceptive awareness, effectively complementing the VSI’s emphasis on visceral sensitivity.

#### 2.3.1. VSI

The Visceral Sensitivity Index (VSI) was developed by Labus et al. in 2004 [1] to evaluate specific anxiety related to the gastrointestinal tract (GSA). The questionnaire consists of 15 items and assesses concern, fear, vigilance, sensitivity, avoidance, as well as cognitions and behaviors related to gastrointestinal aspects, often accompanying misperceptions and misjudgments of bodily sensations. Items are rated on a six-point Likert scale, ranging from 1 (strongly agree) to 6 (strongly disagree). Subsequently, the numbers indicated by the patient for each item are evaluated by assigning a score from 0 to 5. The overall VSI score ranges from 0 to 75, with higher scores indicating more severe GSA. Given the total score, a value greater than 37.5 has been chosen to indicate an increased VSI in individuals [1,2].

#### 2.3.2. MAIA

The Multidimensional Assessment of Interoceptive Awareness (MAIA), developed by Mehling et al. in 2012 [13], is a self-report questionnaire comprising 32 items on a 5-point Likert scale from 0 = never to 5 = always. The questionnaire development was funded by the National Institute of Health (NCCAM/NCCIH) and is in the public domain. Its copyright is with the University of California, San Francisco, and the questionnaire is available in 30 languages (https://osher.ucsf.edu/research/maia; Accessed: 3 May 2024). In this present study, the Italian version was used.

The MAIA provides a multidimensional profile of interoceptive sensitivity, including the following eight subscales:

1. Noticing: awareness of uncomfortable, comfortable, and neutral bodily sensations.
2. Not distracting: tendency to ignore or distract oneself from sensations of pain or discomfort.
3. Not worrying: emotional distress or worry associated with sensations of pain or discomfort.
4. Attention regulation: the ability to sustain and control attention to bodily sensations.
5. Emotional awareness: awareness of the connection between bodily sensations and emotional states.
6. Self-regulation: the ability to regulate psychological discomfort through attention to bodily sensations.
7. Body listening: active listening to the body for understanding.
8. Trusting: experiencing one’s own body as safe and reliable.

Higher scores on the MAIA indicate greater interoceptive sensitivity. In this present study, items related to Interoceptive Awareness obtained an alpha value = 0.86.

### 2.4. Statistical Analysis

The data were analyzed using SPSS 27.0 software (SPSS Inc., Chicago, IL, USA). Continuous variables were reported using mean (M) and standard deviation (SD), while categorical variables were presented as frequencies and percentages.

To test the VSI-IT factor structure, an Exploratory Factor Analysis (EFA) was performed. The EFA was conducted by choosing the number of factors by visually inspecting a scree plot and, by parallel analysis, comparing simulated and observed eigenvalues across multiple solutions (number of factors). The EFA was performed using the *oblimin* rotation method, which would allow for factors to be correlated. The Cronbach’s alpha of each factor was also computed. Following the retrieval of the optimal factorial solution by EFA, a secondary analysis by Confirmatory Factor Analysis (CFA) ensued in order to estimate additional measures of goodness of fit.

To test VSI-IT sensitivity and discriminant validity, we performed the one-way ANOVA between subjects with a comparison for multiple groups, adopting a post hoc Bonferroni-corrected test between groups.

To test the concurrent validity of VSI-IT, correlations between continuous variables were assessed using the Pearson correlation coefficient.

## 3. Results

### 3.1. Factor Structure, Validity, and Reliability of VSI-IT

According to EFA, a one-factor solution was estimated as the optimal factor structure for the collected data. The Kaiser–Meyer–Olkin Measure of Sampling Adequacy (KMO) for collected data (15 items of VSI-IT) was 0.961, far above the acceptable minimum of 0.5, indicating that the data sampling for factor analysis was more than adequate. Bartlett’s test of sphericity, with an approximate chi-square value of 5339.394 and *p* < 0.001, confirmed that a factorial solution for these 15 items was supported. Furthermore, the single-factor solution showed excellent reliability (Cronbach’s alpha 0.949).

The one-factor solution was confirmed by the CFA. The evaluated model consisted of a single latent factor with fifteen observed variables (items of the VSI-IT). After an initial assessment, no significant adjustments were necessary, as the initial model already showed a good fit to the data. The Comparative Fit Index (CFI), Tucker–Lewis Index (TLI), and Standardized Root Mean Square Residual (SRMR) scores were 0.909, 0.894, and 0.046, respectively. These scores are close to or above the generally acceptable level of 0.90 for CFI and TLI, while below the upper threshold of 0.08 for SRMR, indicating that the computed model was reasonably able to capture the underlying factor structure of the variables, while also indicating that the variance explained by the model was significantly higher than random [14,15].

All items showed a factor loading over 0.5 (Table 2). This indicates that each question within the index is closely linked to anxiety related to gastrointestinal symptoms, a central aspect of IBS and IBD. In particular, items with higher factor loadings (exceeding 0.8), such as Item 3 “I often worry about belly problems” and Item 4 “I have difficulty enjoying myself because I can’t take my mind off belly discomfort”. reflect deep and persistent concerns regarding abdominal discomfort and its impact on daily life. At the same time, even items with slightly lower factor loadings, like Item 10, “I am constantly aware of the feeling I have in my belly”. represent relevant aspects of specific anxiety for gastrointestinal symptoms, indicating high awareness and concern for bodily sensations. Furthermore, the percentage of variance explained by the single extracted component, which is significative (approximately 59%), confirms that the VSI is effective in capturing most of the anxiety related to gastrointestinal symptoms in a single construct.

### 3.2. Sensitivity and Discriminant Validity of the VSI-IT

#### 3.2.1. The Ability of the VSI-IT to Discriminate between Pathological Conditions

The analysis of differences between patient groups with various gastrointestinal conditions and healthy controls shows significant results (Table 3 and Table 4). Healthy controls markedly differ from patients with irritable bowel syndrome, Crohn’s disease, and ulcerative colitis, with the latter exhibiting significantly higher levels of visceral sensitivity, indicating a stronger impact of these conditions on anxiety-related visceral symptoms. This difference remains statistically significant even after applying the Bonferroni correction, known for its strict control of Type I errors in multiple analyses (ANOVA between group sums of squares = 65,044.587; df = 4; F = 66.291; *p* < 0.001).

**Figure 1:**
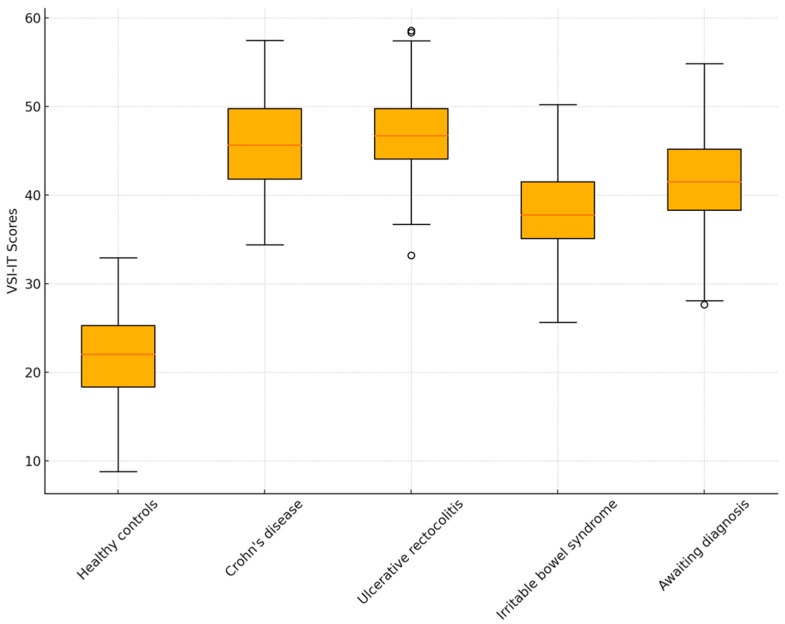
Boxplot VSI-IT between groups. Legend: The x-axis represents the total VSI-IT score (range 0–75); the y-axis represents the comparison groups, divided according to diagnosis (healthy controls; Crohn’s disease; ulcerative colitis; irritable bowel syndrome; subject awaiting for a formal diagnosis).

Post hoc comparison between patients with irritable bowel syndrome and other patient groups did not reveal statistically significant differences before applying the Bonferroni correction, suggesting a similar level of visceral sensitivity among these groups (see Figure 1). This changes when irritable bowel syndrome is compared with ulcerative colitis, where the difference becomes significant but loses significance after adjustment. When comparing groups awaiting diagnosis (“other”) with those affected by Crohn’s disease or ulcerative colitis, visceral sensitivity differences are not statistically significant, indicating that visceral sensitivity among these conditions may not vary substantially. Similarly, the direct comparison between Crohn’s disease and ulcerative colitis shows no significance, suggesting that these two disorders share similar aspects in terms of how patients perceive and react to gastrointestinal symptoms.

#### 3.2.2. The Ability of the VSI-IT to Discriminate Based on the Severity of Symptoms

**Figure 2:**
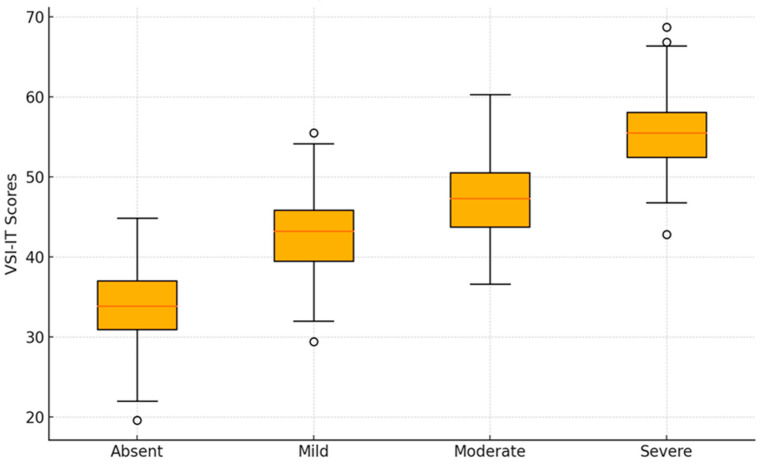
Boxplot of VSI-IT according to symptom severity. Legend: the x-axis represents the total VSI-IT score (range 0–75); the y-axis represents the comparison groups, divided according to the symptoms severity (absent, mild, moderate, severe).

Table 5 and Table 6 show the results of the one-way ANOVA between subjects with a comparison for multiple groups, adopting a post hoc Bonferroni-corrected test, used to assess whether there are significant differences in visceral sensitivity among patient groups with different symptom severity levels: absent, mild, moderate, and severe. In terms of discriminant validity, which refers to the ability of an instrument to distinguish between groups that should differ based on the construct the instrument intends to measure, the graph (Figure 2) suggests that the VSI has a certain ability to discriminate between groups based on symptom severity (F = 17.03; df(3); mean of squares = 3868.91; *p* > 0.001). However, the presence of outliers, especially in the group with severe symptoms, might also indicate that factors other than symptom severity could influence visceral sensitivity, such as anxiety or other psychological factors. In conclusion, the VSI appears to have a certain degree of discriminant validity concerning symptom severity, but the degree of overlap between groups suggests that further research may be needed to explore the complexity of this construct and to better understand the interactions between visceral sensitivity and other psychological or physical factors.

### 3.3. Concurrent Validity of VSI-IT

The correlation table (Table 7) shown in the image provides Pearson correlation coefficients between visceral sensitivity and various psychological constructs, such as attentional ability, emotional regulation, and bodily awareness (MAIA). The concurrent validity of a measurement instrument refers to its ability to significantly correlate with other tests that are theoretically connected to the same construct. Specifically, “Noticing” has a moderate positive correlation with visceral sensitivity (r = 0.277. *p* < 0.001), suggesting that increased attention or awareness of GI symptoms is associated with higher visceral sensitivity. “Not-worrying” shows a moderate negative correlation (r = −0.414. *p* < 0.001), suggesting that the ability not to excessively worry is associated with lower visceral sensitivity. “Attention regulation” has a negative correlation (r = −0.142. *p* = 0.001), which may indicate that increased attention regulation reduces visceral sensitivity. “Emotional awareness” is positively correlated (r = 0.235. *p* < 0.001), meaning that greater awareness of one’s emotions is associated with higher visceral sensitivity. “Self-regulation” shows a weak negative correlation (r = −0.135. *p* = 0.003), suggesting that the ability to self-regulate might have a moderating effect on visceral sensitivity. Finally, the component “Trusting” exhibits a negative correlation with VSI (r = −0.302. *p* < 0.001), implying that greater trust in one’s bodily sensations is associated with lower visceral sensitivity.

## 4. Discussion

This study aimed to examine the psychometric characteristics of the VSI-IT in a sample of Italian-speaking patients with IBS and IBD. The current results show that the translated version of the VSI may reach sufficient internal and external validity. Factor analysis and model fit indices revealed a potential uni-dimensionality for the construct under evaluation, with all 15 items showing high factor loadings, indicating adequate internal validity. On the other hand, the correlations between VSI scores and gastroenterological conditions confirmed the external and convergent validity of the instrument, capable of adequately addressing gastrointestinal-specific and interoceptive complaints.

The VSI-IT also showed sufficient discriminant validity since it resulted in being sensitive in discriminating patients with IBS, Crohn’s disease, and ulcerative rectocolitis from healthy controls. Interestingly, the VSI-IT also discriminated between patients awaiting formal diagnosis from healthy controls. The potential to discriminate patients from controls seems in line with what was previously observed by Trieschmann [2]. In the current results, however, VSI-IT was also able to stratify patients according to symptom severity.

Patients with IBD were previously observed to exhibit high GSA, correlated with a decrease in health-related quality of life (HRQOL). A decrease in HRQOL can negatively impact treatment compliance and long-term disease outcomes. It is believed that IBS symptoms are influenced by both external stressors, such as major life events, and internal stressors, such as excessive conditioned reactivity [16,17]. Indeed, the existence of some outliers, especially among patients with more severe symptoms, suggests that other factors, biological or psychological in nature, may influence visceral sensitivity. Nonetheless, the consistency of the observed differences suggests that the VSI could also be a sensitive indicator of variations in anxiety related to gastrointestinal symptoms [18].

Patients with non-functional gastrointestinal disorders (chronic IBD, cancer) might achieve a relatively low score on the VSI despite the significant severity of symptoms, while patients with other functional gastrointestinal disorders (such as functional dyspepsia or non-cardiac chest pain, and especially those with multiple disorders) might obtain a high score due to generalized anxiety about gastrointestinal sensations [18]. VSI may thus be a valid and sensitive tool suitable for assessing also specific anxiety related to gastrointestinal symptoms, applicable in both clinical practice and research to enhance the understanding and management of IBD, IBS, and other related conditions.

The current findings also suggest that the assessment of GSA during the diagnostic and treatment phase of IBS and IBD may be warranted, potentially allowing clinicians to quantify and monitor the progression or improvement of the disease. From a clinical perspective, in fact, the current results suggest that aspects of emotional and bodily awareness [7,8,9,19], as well as the ability to manage concerns and attention, are correlated with visceral sensitivity. These results support what was previously shown by Atanasova and colleagues [20].

The correlations between the Multidimensional Assessment of Interoceptive Awareness (MAIA) subscales and the Visceral Sensitivity Index showed insightful associations in how individuals with IBD and IBS perceive and react to internal bodily signals [13,21]. For instance, the positive correlation with the “Noticing” dimension indicates that individuals more attuned to bodily sensations also report greater visceral sensitivity, highlighting a heightened worry about internal bodily states.

Conversely, the negative correlation with “Not-worrying” suggests that patients who exhibit less emotional distress or worry in response to pain or discomfort tend to perceive lower visceral sensitivity, possibly reflecting a coping mechanism that dampens the perception of internal discomfort.

Similarly, “Trusting” one’s body, associated with a moderate negative correlation, implies that viewing the body as safe and reliable can reduce sensitivity to visceral signals, potentially offering a psychological buffer against distress. On the contrary, not trusting one’s body could have the opposite effect. If a person perceives their body as unsafe or unreliable, they might experience increased sensitivity to visceral signals. This hypersensitivity can amplify the perception of discomfort or pain, making the person more vulnerable to psychological stress related to unpleasant bodily sensations.

These findings underscore the interplay between cognitive, emotional, and sensory aspects of interoceptive awareness, influencing how people affected by IBD and IBS experience their internal bodily experiences.

### Limitations

There are several limitations to this study that need to be emphasized. First, the diagnoses were self-reported. The groups were not balanced in terms of the number of subjects per group. Additional studies with a more balanced sample size and a broader distribution of various pathologies within IBD would be necessary to draw meaningful conclusions about VSI differences concerning IBD disease types. Additionally, subjects awaiting diagnosis present a very small sample size, which should be improved in the future. No patients with non-binary gender identity were here enrolled. The potential for a gender perspective on GSA might be the focus of future research applications.

The diagnostic sensitivity and specificity of the VSI in patients awaiting diagnosis could also represent a novel avenue for future research, with significant potential for real-world applications. In fact, patients often experience symptoms long before they receive a definitive diagnosis due to the complexity and overlap of gastrointestinal symptoms. Using the VSI could reduce the time between symptom onset and diagnosis, reducing the burden of care and addressing the neglected needs of these patients.

## 5. Conclusions

These results indicate that the Italian version of the VSI is a reliable and valid tool for measuring GSA in IBD and IBS, as well as within healthy controls. Internal consistency is appropriate, and the sample adequacy and item correlation are ideal for factor analysis, confirming the robustness of the Italian VSI’s structure. This makes the instrument particularly useful for research and clinical practice in the context of IBS and IBD in Italian-speaking patients [22,23,24].

Understanding and managing visceral sensitivity through the VSI can lead to a significant improvement in the quality of life for patients [25]. This tool can be crucial for optimal management of gastrointestinal conditions, providing patients and their physicians with a valuable resource to address the complexities and challenges associated with such disorders.

## Figures and Tables

**Table 1:** Sociodemographic characteristics of the sample, divided by diagnosis.

*Variable*	*Category*	*CD* (n = 111)	*UC* (n = 180)	*IBS* (n = 34)	*HC* (n = 164)
***Gender***	Males	4.72%	5.13%	2.05%	9.86%
	Females	18.07%	31.83%	4.93%	23.41%
	Other	0.00%	0.00%	0.00%	0.21%
***Age Group***	18–30 years	6.37%	14.78%	3.49%	23.20%
	31–45 years	10.06%	14.37%	1.44%	3.70%
	46–60 years	5.54%	7.19%	1.23%	4.11%
	61–75 years	0.82%	0.62%	0.41%	2.46%
	76–85 years	0.00%	0.00%	0.21%	0.00%
***Region of Residence***	North	8.62%	16.43%	0.21%	3.08%
	Center	4.93%	6.57%	0.41%	1.64%
	South	4.93%	8.62%	4.93%	21.36%
	Islands	4.31%	5.34%	1.44%	7.39%
***Education Level***	Elementary	0.00%	0.21%	0.00%	0.00%
	Lower Secondary	3.49%	1.64%	0.21%	2.26%
	Upper Secondary	11.29%	17.66%	3.70%	18.89%
	Diploma	0.41%	0.41%	0.00%	0.41%
	Bachelor’s Degree	4.11%	8.01%	1.85%	8.21%
	Postgraduate	2.87%	7.39%	1.23%	3.70%
***Occupational Status***	Student	4.31%	6.57%	3.08%	20.74%
	Employee	6.16%	15.81%	1.44%	4.72%
	Freelancer	2.67%	4.52%	0.82%	2.05%
	Worker	3.08%	2.87%	0.62%	1.23%
	Housewife	4.11%	3.29%	0.62%	2.05%
	Retired	0.41%	0.21%	0.21%	0.82%
***Marital Status***	Single	6.78%	12.12%	3.08%	18.89%
	Married	9.65%	14.17%	2.46%	10.06%
	Cohabiting	4.93%	9.65%	0.82%	2.87%
	Separated	1.03%	0.41%	0.21%	0.21%
	Divorced	0.41%	0.62%	0.21%	1.23%
	Widowed	0.00%	0.00%	0.21%	0.21%

Legend: CD = Crohn’s disease; UC = ulcerative colitis; IBS = irritable bowel syndrome; HC = healthy controls.

**Table 2:** Factor loadings of the single item with the scale, Exploratory Factor Analysis (EFA).

VSI-IT Item	Item Factor Loading
Item 1—I worry that whenever I eat during the day, bloating and distension in my belly will get worse/*Temo che ogni volta che mangio durante il giorno, il gonfiore e la distensione della pancia peggioreranno.*	0.67
Item 2—I get anxious when I go to a new restaurant/*Divento ansioso quando vado in un nuovo ristorante.*	0.74
Item 3—I often worry about problems in my belly/*Mi preoccupo spesso per problemi alla pancia.*	0.83
Item 4—I have a difficult time enjoying myself because I cannot get my mind off of discomfort in my belly/*Ho difficoltà a divertirmi perché non riesco a distogliere la mente dal disagio della pancia.*	0.83
Item 5—I often fear that I won’t be able to have a normal bowel movement/*Spesso temo di non riuscire ad avere un normale movimento intestinale.*	0.82
Item 6—Because of fear of developing abdominal discomfort, I seldom try new foods/*A causa della paura di sviluppare disturbi addominali, raramente provo cibi nuovi.*	0.78
Item 7—No matter what I eat, I will probably feel uncomfortable/*Qualunque cosa mangi, probabilmente mi sentirò a disagio.*	0.80
Item 8—As soon as I feel abdominal discomfort, I begin to worry and feel anxious/*Non appena sento fastidio addominale comincio a preoccuparmi e a sentirmi ansioso.*	0.83
Item 9—When I enter a place I haven’t been before, one of the first things I do is to look for a bathroom/*Quando entro in un posto dove non sono mai stato, una delle prime cose che faccio è cercare un bagno.*	0.71
Item 10—I am constantly aware of the feelings I have in my belly/*Sono costantemente consapevole delle sensazioni che ho nella mia pancia.*	0.58
Item 11—I often feel discomfort in my belly could be a sign of a serious illness/*Sento spesso che il fastidio alla pancia potrebbe essere segno di una grave malattia.*	0.76
Item 12—As soon as I awake, I worry that I will have discomfort in my belly during the day/*Non appena mi sveglio, temo che avrò fastidio alla pancia durante il giorno.*	0.83
Item 13—When I feel discomfort in my belly, it frightens me/*Quando sento disagio nel mio ventre, mi spavento.*	0.76
Item 14—In stressful situations, my belly bothers me a lot/*In situazioni stressanti, la mia pancia mi dà molto fastidio.*	0.72
Item 15—I constantly think about what is happening inside my belly/*Penso costantemente a ciò che sta accadendo nella mia pancia.*	0.84

Legend: Extraction Method: Principal Component Analysis. Applied Rotation Method: oblimin. One component extracted. Eigenvalues for Factor 1 = 8.845. Total of variance explained = 58.964%.

**Table 3:** Descriptives of VSI-IT in various pathological conditions.

	N	Mean	Std. Deviation	Std. Error	95% CI	
					Lower Bound	Upper Bound
Healthy controls	164	21.60	14.90	1.16	19.29	23.90
Crohn’s disease	111	45.53	15.65	1.48	42.58	48.47
Ulcerative rectocolitis	180	47.06	16.31	1.21	44.66	49.46
Irritable bowel syndrome	34	38.94	16.70	2.86	33.11	44.76
Awaiting diagnosis	11	41.36	11.86	3.57	33.39	49.33

**Table 4:** Multiple comparison post hoc test between pathological conditions.

Sample 1	Sample 2	Mean Difference (I-J)	Std. Error	Sig.	95% C.I.	
					Lower Bound	Upper Bound
Healthy controls	Crohn’s disease	−23.93 *	1.92	**<0.001**	−29.36	−18.49
	Ulcerative rectocolitis	−25.46 *	1.69	**<0.001**	−30.24	−20.69
	Irritable bowel syndrome	−17.33 *	2.95	**<0.001**	−25.66	−9.01
	Awaiting diagnosis	−19.76 *	4.87	**<0.001**	−33.51	−6.00
Crohn’s disease	Healthy controls	23.93 *	1.92	**<0.001**	18.49	29.36
	Ulcerative rectocolitis	−1.53	1.89	1.000	−6.86	3.79
	Irritable bowel syndrome	6.59	3.06	0.323	−2.06	15.24
	Awaiting diagnosis	4.16	4.95	1.000	−9.79	18.12
Ulcerative rectocolitis	Healthy controls	25.46 *	1.69	**<0.001**	20.69	30.24
	Crohn’s disease	1.53	1.89	1.000	−3.79	6.86
	Irritable bowel syndrome	8.12	2.92	0.057	−0.13	16.38
	Awaiting diagnosis	5.70	4.86	1.000	−8.01	19.41
Irritable bowel syndrome	Healthy controls	17.33 *	2.95	**<0.001**	9.01	25.66
	Crohn’s disease	−6.59	3.06	0.323	−15.24	2.06
	Ulcerative rectocolitis	−8.12	2.92	0.057	−16.38	0.13
	Awaiting diagnosis	−2.42	5.43	1.000	−17.74	12.89
Awaiting diagnosis	Healthy controls	19.76 *	4.87	**<0.001**	6.00	33.51
	Crohn’s disease	−4.16	4.95	1.000	−18.12	9.79
	Ulcerative rectocolitis	−5.70	4.86	1.000	−19.41	8.01
	Irritable bowel syndrome	2.42	5.43	1.000	−12.89	17.74

* The mean difference is significant at the 0.05 level.

**Table 5:** Mean comparisons of symptoms severity.

W	N	Mean	Std. Deviation	Std. Error	95% C.I.	
					Lower Bound	Upper Bound
Absent	54	34.79	17.85	2.42	29.92	39.66
Mild	94	43.57	16.43	1.69	40.20	46.94
Moderate	144	47.82	13.15	1.09	45.66	49.99
Severe	41	55.73	14.13	2.20	51.26	60.19

Legend: the significance level is 0.050. Significance values have been adjusted by the Bonferroni correction for multiple tests.

**Table 6:** Multiple comparison post hoc test, according to symptoms severity.

Symptoms Severity		Mean Difference (I-J)	Std. Error	Sig.	95% Confidence Interval	
					Lower Bound	Upper Bound
Absent	Mild	−8.77 *	2.57	**0.004**	−15.60	−1.94
	Moderate	−13.03 *	2.40	**<0.001**	−19.41	−6.64
	Severe	−20.93 *	3.12	**<0.001**	−29.22	−12.64
Mild	Absent	8.77 *	2.57	**0.004**	1.94	15.60
	Moderate	−4.25	1.99	0.205	−9.55	1.05
	Severe	−12.15 *	2.82	**<0.001**	−19.64	−4.66
Moderate	Absent	13.03 *	2.40	**<0.001**	6.64	19.41
	Mild	4.25	1.99	0.205	−1.05	9.55
	Severe	−7.90 *	2.66	**0.020**	−14.98	−0.82
Severe	Absent	20.93 *	3.12	**<0.001**	12.64	29.22
	Mild	12.15 *	2.82	**<0.001**	4.66	19.64
	Moderate	7.90 *	2.66	**0.020**	0.82	14.98

* The mean difference is significant at the 0.05 level.

**Table 7:** Correlations between MAIA and VSI-IT.

Variable	Visceral Sensitivity Index
Noticing	0.277 **
Not distracting	−0.044
Not worrying	−0.414 **
Attention regulation	−0.142 **
Emotional awareness	0.235 **
Self-regulation	−0.135 *
Body listening	0.056
Trusting	−0.302 **

Legend: ** *p* < 0.001; * *p* < 0.003.

## Data Availability

Data will be made available by the corresponding author upon reasonable request.

## Appendix A

Visceral Sensitivity Index—Italian Version (VSI-IT) © 2024 by Rizzo, Mautone, Sitibondo, et al.

	Pienamente d’accordo	D’accordo	Parzialmente d’accordo	Parzialmente in disaccordo	In disaccordo	Fortemente in disaccordo
1. Temo che ogni volta che mangio durante il giorno, il gonfiore e la distensione della pancia peggioreranno.	□	□	□	□	□	□
2. Divento ansioso quando vado in un nuovo ristorante.	□	□	□	□	□	□
3. Mi preoccupo spesso per problemi alla pancia.	□	□	□	□	□	□
4. Ho difficoltà a divertirmi perché non riesco a distogliere la mente dal disagio della pancia.	□	□	□	□	□	□
5. Spesso temo di non riuscire ad avere un normale movimento intestinale.	□	□	□	□	□	□
6. A causa della paura di sviluppare disturbi addominali, raramente provo cibi nuovi.	□	□	□	□	□	□
7. Qualunque cosa mangi, probabilmente mi sentirò a disagio.	□	□	□	□	□	□
8. Non appena sento fastidio addominale comincio a preoccuparmi e a sentirmi ansioso.	□	□	□	□	□	□
9. Quando entro in un posto dove non sono mai stato, una delle prime cose che faccio è cercare un bagno.	□	□	□	□	□	□
10. Sono costantemente consapevole delle sensazioni che ho nella mia pancia.	□	□	□	□	□	□
11. Sento spesso che il fastidio alla pancia potrebbe essere segno di una grave malattia.	□	□	□	□	□	□
12. Non appena mi sveglio, temo che avrò fastidio alla pancia durante il giorno.	□	□	□	□	□	□
13. Quando sento disagio nel mio ventre, mi spavento.	□	□	□	□	□	□
14. In situazioni stressanti, la mia pancia mi dà molto fastidio.	□	□	□	□	□	□
15. Penso costantemente a ciò che sta accadendo nella mia pancia.	□	□	□	□	□	□
